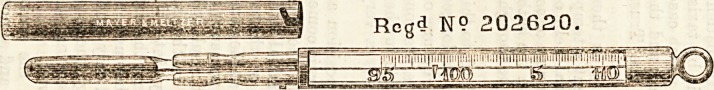# New Drugs, Appliances, and Things Medical

**Published:** 1893-02-04

**Authors:** 


### NEW DRUGS, APPLIANCES, AND THINGS MEDICAL.

[All preparations, appliances, novelties, etc., of which a notice is desired, should be sent for the Editor, to care of The Manager, 140, Strand, London, W.C.]

### THE NEWTON-NIXON THERMOMETER CASE.

Messrs. Meyer and Meltzer, under the direction of Mr. Nixon, the energetic secretary of University College Hospital, have offered to the medical and nursing profession a new thermometer case. The advantages they claim for their invention are mainly as follows: (1) After unscrewing the top the thermometer need not be further removed from the case, and the temperature may be taken with the tube still lying *in situ*, the scale being read through a slit in the metal case. (2) A considerable saving of time in unscrewing the top, only one movement being necessary instead of many, as is usually the case. (3) If the temperature be taken in the mouth, the patient instead of biting the fragile glass tube bites the resistant metal case. A reference to the illustration will make these points clear. These advantages are substantial if they are real. Whether, however, the apparently ingenious contrivance of Mr. Nixon for the protection of the thermometer in use and while being carried about will effect any appreciable reduction in the annual breakage role of thermometers in hospitals and like institutions, time alone can prove; but after a short trial of the Newton-Nixon case we feel we can conscientiously recommend its adoption by all who are interested in the reduction of the annual loss incurred by the breakage of thermometers. The disadvantages are few and comparatively insignificant, but a few points we should like to mention. In the first place, any time that is saved in unscrewing the top is more than compensated for by additional time occupied in cleaning the case after use. And even the drying is certainly a matter of difficulty and time. In children's wards, where the metal protection would probably effect the largest saving if the temperature were taken in the mouth, the method of taking the temperature in the rectum is acquiring daily a larger following, and in this case a metal case would be clearly inapplicable. Also we very much doubt whether the thermometer could be safely carried on a chain or chatelain. A smart knock would probably break the thermometer in any sort of case, except indiarubber.

## Figures and Tables

**Figure f1:**